# Er:YAG Laser Dental Treatment of Patients Affected by Epidermolysis Bullosa

**DOI:** 10.1155/2014/421783

**Published:** 2014-11-05

**Authors:** Angela Galeotti, Vincenzo D'Antò, Tina Gentile, Alexandros Galanakis, Simona Giancristoforo, Roberto Uomo, Umberto Romeo

**Affiliations:** ^1^Dentistry Unit, Department of Pediatric Surgery, Bambino Gesù Children's Hospital, IRCCS Ospedale Pediatrico Bambino Gesù, Piazza S. Onofrio No. 4, 00165 Rome, Italy; ^2^Dermatology Unit, Department of Pediatric Medicine, Bambino Gesù Children's Hospital, IRCCS Ospedale Pediatrico Bambino Gesù, Piazza S. Onofrio No. 4, 00165 Rome, Italy; ^3^Department of Oral and Maxillofacial Sciences, “Sapienza” University of Rome, Via Caserta No. 6, 00161 Rome, Italy

## Abstract

*Aim*. The purpose of this study was to evaluate the efficacy of Er:YAG laser used for treating hard dental tissue in patients with epidermolysis bullosa (EB). *Methods*. We report two cases of EB in which an Er:YAG laser was used for conservative treatments. In the first case, the Er:YAG laser (2,940 *μ*m, 265 mJ, 25 Hz) was used to treat caries on a deciduous maxillary canine in an 8-year-old male patient affected by dystrophic EB. In the second case, we treated a 26-year-old female patient, affected by junctional EB, with generalized enamel hypoplasia, and an Er:YAG laser (2,940 *μ*m, 265 mJ, 25 Hz) was used to remove the damaged enamel on maxillary incisors. *Results*. The use of the Er:YAG laser, with the appropriate energy, was effective in the selective removal of carious tissue and enamel hypoplasia. During dental treatment with the Er:YAG laser, patients required only a few interruptions due to the absence of pain, vibration, and noise. *Conclusions*. Laser treatment of hard dental tissues is a valuable choice for patients affected by EB since it is less invasive compared to conventional treatment, resulting in improved patient compliance.

## 1. Introduction

Epidermolysis bullosa (EB) is a rare genodermatosis with marked fragility of the skin and mucous membranes. The main aspect of this genetic disorder is the occurrence of vesiculobullous lesions spontaneously or as a response of a thermal or mechanical trauma. The incidence varies between 1 : 50000 and 1 : 500000 live births [1]. EB affects all racial and ethnic groups, with no gender predominance, often manifesting at birth or during the first years of life [2, 3]. Dental treatment plays a key role in the multidisciplinary care of patients with EB, because it allows mastication and nutrition and avoids oral infection and esophageal soft-tissue damage [4]. The severity of EB may influence the approach to the dental treatment. The classification of inherited EB, based on the level of blistering within the dermal-epidermal junction, includes four broad categories: EB simplex (EBS), junctional EB (JEB), dystrophic EB (DEB), and Kindler syndrome [5]. EBS is characterized by intradermal blistering of the skin and mucous membranes that usually heal without scarring. Dental treatment does not require any modifications [6]. Lesions of JEB are located in the lamina lucida or epidermal-dermal interface. Dental management requires a few modifications, including careful manipulation and absence of adhesive contact [6]. DEB is inherited in both dominant and recessive forms, caused by mutation in type VII collagen gene and it is characterized by blistering in the underlying connective tissue [3]. The worst form of EB is the recessive subtype (RDEB), marked by a high tendency of blistering and skin ulceration. Dental management of patients affected by RDEB requires lubrication of oral tissues, gloves, and instruments to avoid adherence and formation of bullae. The pressure must be gentle when handling the tissue. The suction tip should lean on hard tissues to avoid epithelial sloughing. Blood- and fluid-filled bullae that appear during dental treatment must be drained with scissors or a sterile needle to avoid spreading [6]. Kindler syndrome, marked by a mixed level of cleavage, is the fourth category and its dental approach is focused on limiting mucosal sloughing [6].

The use of laser technology in dentistry has increasingly gained interest since 1990. The Erbium:Yttrium-Aluminum Garnet (Er:YAG) laser is used to manage hard dental tissues because of the affinity of its wavelength for water and hydroxyapatite. It has been shown that employing the Er:YAG laser in conservative dentistry can be a valuable alternative to conventional instruments, such as the turbine and the micromotor [7], supporting a new concept of modern conservative dentistry based on the adhesive method and a minimally invasive approach. The therapeutic selective ablation of damaged dental structure and the creation of a rough surface with opened dental tubules are obtained avoiding the removal of healthy dental tissue, micro- and macrofractures, temperature increases, and the smear layer formation [7, 8].

The Erbium:Yttrium-Aluminum Garnet (Erbium:YAG) and Erbium Chromium:Yttrium-Scandium-Gallium-Garnet (Er,Cr:YSGG) lasers are emitted in the wavelengths of 2,940 *μ*m and 2,780 *μ*m, respectively, and are well absorbed by biological tissues including enamel and dentin; in fact, these wavelengths match two of the absorption peaks of water [9].

Specifically, the wavelength of the Er:YAG laser (2,940 *μ*m) is indicated for the treatment of hard and soft tissues in which it shows excellent absorption by hydroxyapatite and water, allowing “cold ablation” and the cutting of soft tissues without coagulation effects [10].

The Er:YAG laser can produce a really small spot of ablated tissue, less than 1 mm in diameter for most of the devices, which is smaller than most of the frequently employed rotary instruments. This allows for the very effective and selective removal of pathological tissues [11]. Eberhard et al. [12] revealed that using an Er:YAG laser for caries removal resulted in less dentine loss when compared to conventional rotary instruments in an in vitro study.

The efficacy of lasers in the removal of caries was also proven in vivo, revealing a good capacity of decontamination and an acceptable degree of tolerability by the patients in spite of the longer time required to complete the caries excavation [13]. Similar results were also obtained in vivo for primary teeth in children with a procedure that was judged by 93.8% of the patients involved in the study as “comfortable” regarding pain sensations, when anesthesia was not used [14].

A study comparing pain perception during cavity preparation in patients aged 7 to 12 revealed a better patient compliance, with less pain perception in the laser treated group [15].

Even though a number of in vitro works regarding effects of lasers on enamel and dentin of both primary and permanent teeth have been published, a recent revision on the topic concluded that it is advisable to respect the conventional etching procedures, even if laser etching is achieved [16].

Another concern about laser dentistry is restoration durability. A study from Yazici et al. [17] revealed that laser-prepared cavities restored with composite materials have the same durability of bur-prepared cavities in a two-year period.

In the relevant literature, there are no articles dealing with the treatment of Er:YAG laser on hard dental tissues in patients affected by EB. We present two case reports of EB patients aimed at describing the advantages of using the Er:YAG laser in their dental management.

## 2. Clinical Cases Descriptions

### 2.1. Case 1

An 8-year-old Caucasian boy with Hallopeau-Siemens dystrophic EB was referred to the dentistry unit of Bambino Gesù Children's Hospital. Hallopeau-Siemens type is the most severe form of the dystrophic epidermolysis bullosa and its genetic explanation is mutation of the VII collagen gene [18]. Type VII collagen is the main component of anchoring fibrils, which anchor the epidermis to the dermis. When the production of type VII collagen is changed, minor trauma can cause the formation of blisters due to the separation of two skin layers [18].

The patient reported difficulty in daily oral hygiene because of his reduced mouth opening and limited manual dexterity.

His medical history included chronic anemia, malnutrition, corneal leukoma, dysphagia, esophageal stenosis, recurrent respiratory infections, syndactyly, and absence of the nails and fingers of his hands. At the age of 5, he had surgical correction for syndactyly of both hands. His diet was limited to soft or pureed foods. He had normal cognitive function.

As reported by his parents, he suffered from extensive caries of deciduous teeth and, at the age of 4, he underwent multiple dental extractions of all deciduous molars and lower canines under general anesthesia.

At time of presentation, he had extensive skin lesions in the form of bullae, with some scarring on his extremities, neck, and face and he was unable to open his hands.

**Figure 1:**
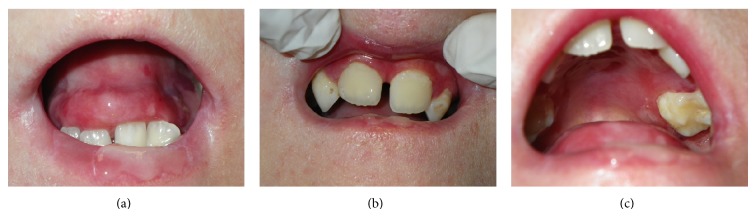
Intraoral aspects in a patient with DEB (microstomia, caries, absence of lingual papillae, and blood- and fluid-filled bullae).

**Figure 2:**
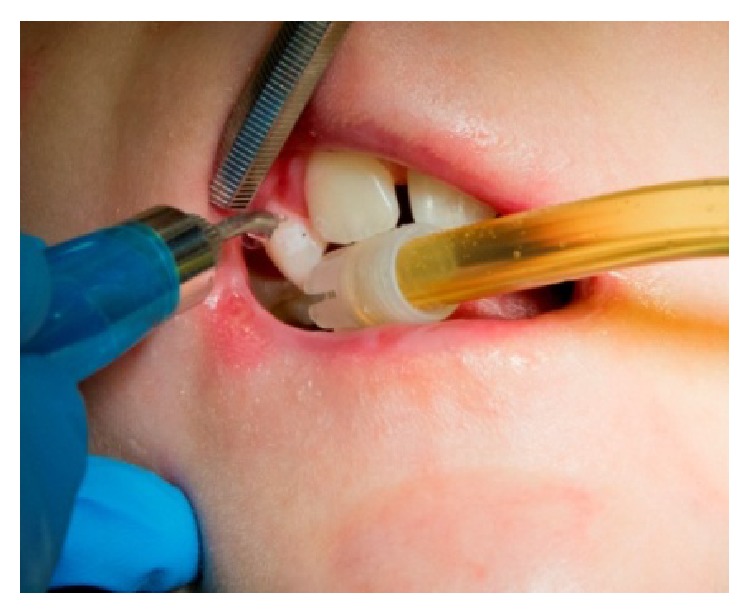
Treatment of hard dental tissues with Er:Yag laser.

The intraoral aspects were ankyloglossia, microstomia, obliteration of vestibule, caries, absence of lingual papillae, and blood- and fluid-filled bullae. The intraoral blisters were various sizes and the gingival tissue was red, edematous, and ulcerated (Figure 1). After intraoral examinations, we decided to treat caries on deciduous maxillary canines using the Er:YAG laser (Hoya ConBio Delight, Sweden & Martina, Padova, Italy) at wavelength = 2,940 *μ*m. Conventional isolation of the operatory field was not possible because of the intraoral features of EB. The patient's lips were lubricated with vaseline/petrolatum and divaricated using the handle of the mirror with gentle compressive movements. The suction tip was leaned against the occlusal tooth surface and the air syringe was used carefully. The Er:YAG laser was used for removing caries at the following parameters: energy = 265 mJ, frequency = 25 Hz, and pulse duration = (fluence was 93 J/cm²). The contact quartz tip was curved at 80° and its diameter measured 600 *μ*m (Figure 2). We selected this contact tip because it allowed us to obtain 90° angle between the laser beam and the tissue. The movement of the Er:YAG laser was slow and continuous over the whole working area with a visual check of the ablating area, in order to remove only infected tissue. The water cooling avoided negative thermal effect and increased the detergent action on the treated tissue. The treated tissue appeared rough and chalky confirming the antimicrobial and decontaminating properties of the Er:YAG laser.

**Figure 3:**
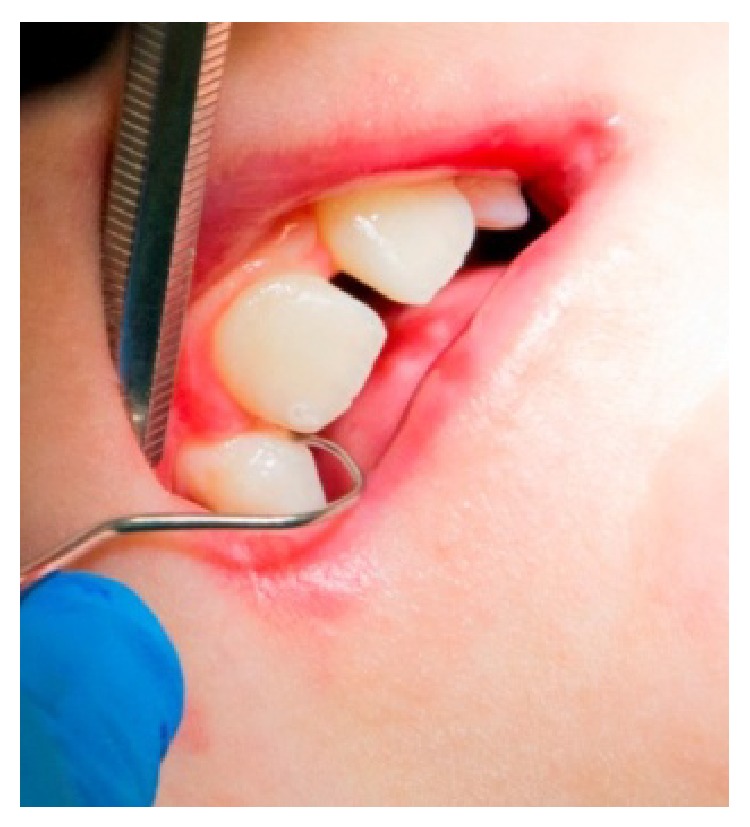
Final result of conservative treatment.

We clinically assessed hard dental tissues by means of a chemical caries detector (Caries Detector, Kuraray Europe Italia, Milan, Italy). The final stage was to perform direct composite reconstructions (Figure 3). During the procedure, the procedure was comfortable because Er:YAG laser avoided vibrations and thermal variations. The use of the Er:YAG laser did not require the anesthesia which could develop iatrogenic blisters. The patient and parents received recommendations for daily oral care. During a follow-up period of 6 months, we clinically evaluated the stability of dental restorations and an improvement of his oral hygiene. In this follow-up recall, the patient stated his satisfaction for dental treatment and absence of postoperative hypersensitivity.

### 2.2. Case 2

**Figure 4:**
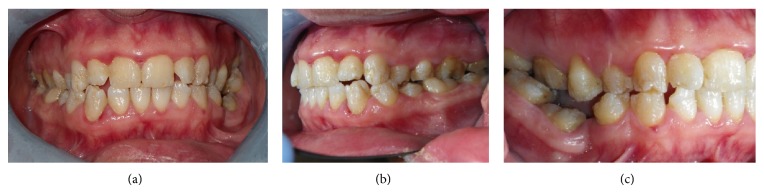
Intraoral aspects of a patient with JEB (generalized enamel hypoplasia).

**Figure 5:**
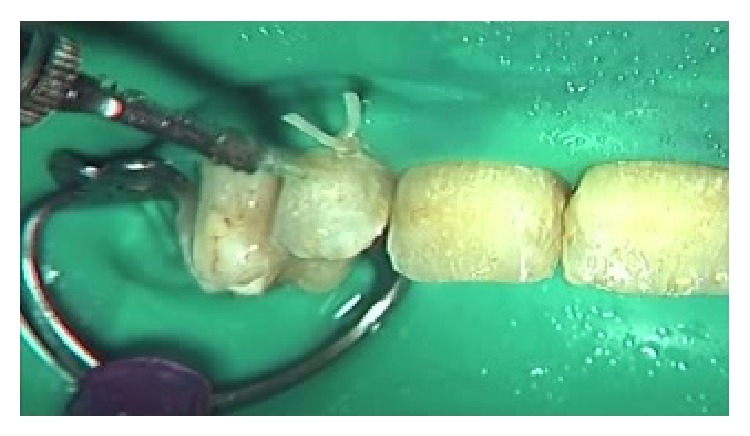
Treatment of the damaged enamel with Er:Yag laser.

**Figure 6:**
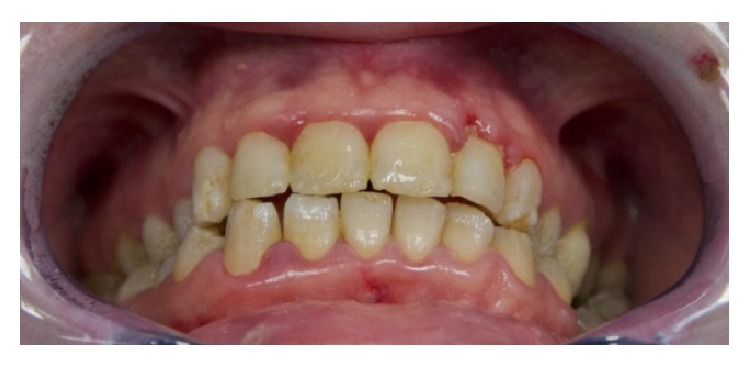
Final result of conservative treatment.

A 26-year-old Caucasian female with junctional EB presented to the Dentistry Unit of Bambino Gesù Children's Hospital for alterations in the structure of her teeth. Her medical history included alopecia, acne, lymphedema, erosion of her scalp, and oligomenorrhoea. At the time of presentation, she had blood- and fluid-filled bullae on her hands and feet and there was absence of nails. The intraoral manifestations were the absence or minimum presence of bullae, edematous gingival tissue, and generalized enamel hypoplasia (Figure 4). We decided to remove the damaged enamel on maxillary incisors using the Er:YAG laser, because the patient complained for esthetic reasons. We used a traditional technique of operatory field isolation with a rubber dam and clamps because her intraoral conditions allowed the use of this tool without negative effects. The parameters used with the Er:YAG laser were 265 mJ and 25 Hz. The contact quartz tip was 80° curved and its diameter was 600 *μ*m, with a fluence of 93 J/cm² (Figure 5). We managed the laser beam which had selective action over a small amount of damaged enamel. The water cooling avoided intraoperative hypersensitivity and increased the detergent action. The effects of the Er:YAG laser were checked clinically and also with chemical caries detector. The final appearance of working area was cratered and irregular and it improved adhesive retention in reconstructive phase. The use of rotating instruments could have removed both damaged and not damaged enamel; in fact, the Er:YAG laser allowed selective ablation of damaged tissue promoting enamel-dentin decontamination and without any signs of thermal damage. Reconstruction of teeth was carried out using template indexes based on a wax-up to build incisal margins. Vestibular surfaces, interproximal emergence profiles, macro- and microsurface textures, and chromatic features were reproduced with a free hand technique (Figure 6). During the procedure, the procedure was interrupted few times upon request of the patient. During the 6-month follow-up period, we clinically evaluated the stability of the dental restorations and the patient stated her satisfaction for esthetic results and the absence of postoperative hypersensitivity.

## 3. Discussion

The two cases presented illustrate many features of dystrophic and junctional EB. Cutaneous findings include blistering, ulcerations, and contractile scars over large body surfaces, cicatricial alopecia, dystrophic nails, and syndactyly. Lesions usually start to appear at birth or within the first 6 months of life and they are frequently associated with chronic blood loss, which may lead to chronic anemia. Extracutaneous findings include the eyes, oral mucosa, teeth, upper and lower gastrointestinal tract, genitourinary tract, trachea, and musculoskeletal system, which can cause systemic complications such as malnutrition, respiratory disorders, and esophageal stricture [19]. The extent of oral involvement varies among the different types of EB. In the mild forms, small blisters (<1 cm in diameter) may develop and heal without scarring. In more severe forms, the continuous process of blister formation and healing changes in the oral architecture. The tongue loses the lingual papillae and becomes bound to the floor of the mouth, which is a condition known as ankyloglossia. Anatomical structures, such as palatal rugae, are ablated. The oral vestibules become obliterated with the soft tissue attachment advancing. The soft tissues defining the oral opening fail to grow normally due to scarring, resulting in a restricted oral aperture (microstomia) [20]. Hard dental tissues can present dental anomalies of number, form, position, and structure (hypoplasia and hypomineralization); these anomalies lead to a high caries risk due to the ingestion of soft, sugary foods and difficulties with oral hygiene [21].

Studies on the chemical composition of enamel from EB patients, in terms of mineral content, carbonate content, protein content, and amino acid composition, have reported essentially normal enamel chemistry in DEB patients, whereas JEB enamel contained a significantly reduced mineral per volume content, which resulted in enamel hypoplasia. Overall, no difference between the mean mineral content of EB teeth and normal controls was observed, although marked alterations in the enamel structure, such as prismatic structure and orientation and surface pitting, were observed in JEB teeth [22].

The two case reports demonstrated a new therapeutic approach to dental treatment in patients with EB. The choice of laser treatment was due to the minimal invasiveness of this tool. The particular features of EB require that extensive use of high speed air spray should be avoided. Therefore, air abrasion was not used, in order to avoid extensive soft tissue disepithelization. Great care was taken in respecting oral mucosa, during both pathological tissue removal and restoration phases. Several reasons support the use of Er:YAG laser in patients with EB. Enamel defects are often found in EB affected patients and inadequate oral hygiene procedures may cause an increased risk of caries, especially in teeth with defective enamel. Frequent follow-up visits are required in order to early detect carious lesions. Preventive measures may be employed (topical fluoroprofilaxis), but when carious lesions are established, treatment should be timely programmed [4–6].

These are the only case reports in which lasers have been used as therapeutic tools in the dental treatment of EB patients. Işeri et al. [23] used CO_2_ laser to perform an excision of fibrous tissues in two patients affected by EB. Indeed, we used Er:YAG laser to treat hard tissue and we wanted to verify how its characteristics could improve the dental management of EB patients. The use of the same parameters of Er:Yag laser in both clinical situations supported positive results of scanning electron microscopy (SEM) analysis for laser-prepared cavities in primary and permanent teeth [24]. During treatment, we noticed that the patients were comfortable and they required few interruptions to rest. The absence of noise, vibrations, and contact from the Er:YAG improved dental management of EB patients, and the absence of negative thermal variations eliminated the need for anesthesia which could develop iatrogenic blisters. EB patients were safely treated because laser beam did not cause problems for adjacent hard and soft tissues.

The minimally invasive approach and the decontaminating effect of the Er:YAG laser simplified the work of the clinician and limited the risk of secondary caries. Furthermore, the clinical-histological effects of the Er:YAG laser made it possible to overcome the limits of traditional dentistry and obtain the best results for EB patients.

## 4. Conclusion

Laser treatment of dental hard tissues is a valid technique for a more comfortable and minimally invasive intervention. It allows for more conservative preparations compared to conventional dental treatment. These characteristics support less use of anesthetic, avoid the development of iatrogenic blisters in EB patients, and ensure the optimal safety of adjacent hard and soft tissues. The Er:YAG laser dental treatment shows improved dental management and positive psychological effects for patients affected by EB.
